# Insomnia

**Published:** 1890-03-22

**Authors:** 


### THE PRACTITIONER'S BOOKSHELF.

(Books for Review should be sent to The EDITOR, The Lodge, Porchester Square, W.)

### INSOMNIA.*

HEALTHY man may be said to pass from one-fourth to one-third of his life in sleep, hence the importance of the loss to him of that power which is practically the daily renewal to him of the forces of life. It is a curious fact that, although in special works on medicine there are chapters on insomnia, there has been no recent complete study of insomnia till Dr. Macfarlane's work appeared.

This is a most thorough work, and one which it is a pleasure to read and to refer to. Dr. Macfarlane has treated the subject from the causal standpoint, and his tables present a most interesting series of studies showing age, sex, and cause. Omitting sleeplessness induced by pain and trivial reasons, 273 cases of insomnia occurred mainly as follows:—

| | | | | |
|---|---|---|---|---|
| 1. Neurasthenia ... | ... 37 | cases or | 13·55 | per cent. |
| 2. Worry ... ... | ... 35 | ,, | 13·18 | ,, |
| 3. Gout ... ... | ... 26 | ,, | 9·52 | ,, |
| 4. Overwork ... | ... 23 | ,, | 8·04 | ,, |
| 5. Menopause ... | ... 18 | ,, | 6·59 | ,, |
| 6. Dyspepsia ... | ... 17 | ,, | 6·22 | , |
| 7. Alcoholism ... | ... 16 | ,, | 5·86 | ,, |
| 8. Senility ... ... | ... 12 | ,, | 4·39 | ,, |

This table represents the experience of three competent observers, and we can take it as accurately showing the causes and numbers in a general run of cases of insomnia.

Dr. Macfarlane, after giving tables and a general idea of the causation of insomnia, then proceeds to take each of the above special headings into review, beginning with Overwork. Speaking in the division labelled "Depressing Emotions," he says: "It is important, in the first place, to ascertain the cause of the worry, so that if it has its origin in some bodily ailment, such as varicocele, the cause may be removed. In many cases, however, the patient seeks advice for insomnia or dyspepsia with troubled sleep, and so far from hinting that it is due to grief, he seeks to conceal his sorrow, and it requires delicacy and tact to overcome reticence, and to obtain a frank confession of the trouble."

"If it is a monetary anxiety, the physician might prove himself a magician if, instead of writing a prescription, he could

* "Insomnia and its Therapeutics." By A. W. Macfarlane, M.D. London: H. K. Lewis, 136, Gower Street. 1890.

give an order on his banker for the amount. Unfortunately few physicians have it in their power to do so, and those who have do not appear to think of it! The physician's powers are limited in cases of anxiety about personal health or that of friends, or when grief depends on bereavement, or, still worse, upon living sources of sorrow. There is one thing, however, the physician has in his power—he can bring a clear and unbiassed mind to bear upon the case, and by judicious advice and counsel he may go a long way towards putting the sufferer into a condition for bearing his trials manfully."

Dr. Macfarlane insists over and over again on the importance of diet. He, with therest of the profession, has found that drugs, like the offensive weapons of warfare, are good to defend and ward off the attacks of the enemy; but the true defensive power lies greatly in the proper feeding and hygienic surroundings of the patient. Many years ago the late Dr. Milner Fothergill said to us: "The most successful physician is he who feeds his patient aright." The commonest question put to the doctor, after he has ascertained what is the matter with the patient, is, "What food shall we give him?" This is the moment when the successful treatment of the case often hangs on the directions of the doctor. Fortunately for us modern science has simplified the question by putting into our hands foods and methods of feeding which our fathers had no idea of, and also we have what is better, a skilled, kind, and attentive helpmeet in the form of the modern trained nurse. We know that, when we see the neat uniform standing by the bedside, we have a subaltern who will carry out our orders in a way that makes success, if not sure, at any rate well deserved.

Dr. Macfarlane gives full and particular directions, under the different causal headings, for the diet and for the appropriate drug treatment.

In speaking of Overwork, he says: "The evils of mental overwork are frequently manifested in children, owing to the pernicious system of *cram* which prevails so extensively at the present day. Their brains require an unusually large amount of sleep for the purpose of supplying the necessary pabulum for growth, as well as for the removal of the products caused by tissue-metamorphosis. This fact presents itself in a striking light when it is remembered that the male brain reaches five-sixths and the female ten-elevenths of its full size at the age of seven years." Who among us has not seen many cases of brain tiredness and general illness caused by that modern Moloch, "compression-education" or cramming? We would here insist especially on the absolute need of rest to school-girls at the critical period of 14—17. It should be a rule without exception, that at the appropriate periods these girls should do no mental work whatever. Head-work done then often leads to a nervous mental condition which takes years to recover from or perhaps never is thoroughly eradicated. "Bed, weakens" is the old-fashioned cry. Under modern conditions of over-pressure, we answer, "Bed, strengthens, for it gives the overwrought system time to recover."

Dr. Macfarlane's directions for the use of narcotics are most judicious, and show a full acquaintance with modern ideas regarding them. He insists on the dangers of acquiring the habitual use of them, but says wisely, that when the patient is perishing for their want he is a poor physician who withholds them because he is afraid of their after effects.

We note that Dr. Macfarlane speaks well of sulphonal as being of great use in certain varieties of insomnia. Here we can corroborate him. We have seen less ill-effects after its use than after any other form of trustworthy hypnotic. Of course, it does not answer in all cases, and it ought *only* to be taken under the advice of a medical man, and this rule is without an exception.

This book is pleasant reading, and, what is of most importance, extremely helpful. The whole volume bears the impress of that "philosophical spirit" which Dr. Clifford Allbutt, in his recent address to medical students, says is characteristic of early academical and university training. In conclusion, we can say, after a careful perusal of this book, that Dr. Macfarlane has succeeded in his task, and we are sure that his work will enable the profession to combat successfully that most trying condition, which the well-chosen motto on the title-page describes in the words of Job: "When I lie down I say, When shall I arise and the night be gone? and I am full of tossings to and fro unto the dawning of the day."

